# An improved Deeplabv3+ semantic segmentation algorithm with multiple loss constraints

**DOI:** 10.1371/journal.pone.0261582

**Published:** 2022-01-19

**Authors:** Yunyan Wang, Chongyang Wang, Huaxuan Wu, Peng Chen

**Affiliations:** 1 School of Electrical and Electronic Engineering, Hubei University of Technology, Wuhan, China; 2 Hubei University of Technology Cooperative Innovation Center of Hubei Province for Efficient Use of Solar Energy, Wuhan, China; University of Bradford, UNITED KINGDOM

## Abstract

Aiming at the problems of low segmentation accuracy and inaccurate object boundary segmentation in current semantic segmentation algorithms, a semantic segmentation algorithm using multiple loss function constraints and multi-level cascading residual structure is proposed. The multi-layer cascaded residual unit was used to increase the range of the network layer receptive field. A parallel network was constructed to extract different depth feature information, then different depth feature information and encoder output features are fused to obtain multiple outputs feature which build multiple losses with the label, thereby constraining the model to optimize the network. The proposed network was evaluated on Cityscapes and CamVid datasets. The experimental results show that the mean Intersection over Union ratio (MIoU) of the proposed algorithm is 3.07% and 3.59% higher than the original Deeplabv3+ algorithm, respectively.

## 1 Introduction

Image segmentation, as an important branch in the field of computer vision, can achieve accurate positioning and identification of object locations while reducing costs, and is widely used in unmanned driving, medical image processing, industrial robots and other fields [1, 2]. In 2015, the Fully Convolutional Neural Network (FCN) was proposed, which made deep learning formally used in the field of semantic segmentation [3]. FCN used VGG Convolutional Neural Network as the backbone Network. After removing all the full connection layers, a deconvolution layer is added at the end of the network to upsample the input image, and the dense pixel prediction results are obtained. Compared with the traditional segmentation algorithm [4], FCN has a great improvement in the segmentation accuracy, but it has poor segmentation effect in the segmentation of small target objects [5]. The problem of FCN is that it only relies on high-level feature information to classify pixels, and lacks the use of low-level feature images with rich detailed information, which leads to rough final segmentation results of the network. Therefore, how to rationally use the underlying feature information to enhance the semantic segmentation effect has become a hot research issue. Researchers have proposed some semantic segmentation models using codec structure, such as U-Net [6], Multi-Path Refinement Networks (Refinenet) and SegNet [7, 8]. The above segmentation model uses the codec structure to supplement the details lost by the model’s lower sampling in the process of up-sampling to improve the segmentation performance of the model [9]. Zhao et al. proposed the Pyramid Scene Parsing Network (PSPNet) semantic segmentation model, which can integrates multi-scale context information through the pyramid scene analysis network to obtain excellent segmentation results [10, 11]. In addition, using the attention mechanism to strengthen the network’s learning and attention to important features is also a way to improve the semantic segmentation effect. Danet uses the dual attention mechanism of space and channel to capture the semantic interdependence in space and channel dimensions, which significantly improves the segmentation effect [12, 13]. In addition, Chen et al. proposed the Deeplab series semantic segmentation model, in which V1 proposed to replace the pooling layer with void convolution to avoid the loss of detailed information and obtain a larger receptive field range. In addition, Conditional Random Field (CRF) was added at the end of the model to optimize the boundary segmentation [14]. In DeepLabV2, a void space pyramid module was proposed to obtain global semantic information at multiple scales [15]. Then, DeepLabV3 and DeepLabV3+ have improved the void space pyramid module again [16, 17] and replaced Inception as a new backbone network, which has become the most effective semantic segmentation algorithm in the current semantic segmentation field [18, 19]. Recently, Fu et al. [13] proposed a dual-focus network for scene segmentation, which can capture rich context dependencies based on self-focus mechanism. Specifically, they attached two types of focus modules to the expanded FCN, which modeled semantic interdependence in FDI. The location notice module selectively aggregates the features of each location by a weighted sum of the features of all locations. Huo et al. [20] proposed the concept of bar pooling and used horizontal average pooling and vertical average pooling to model global information in an attempt to approach the long-term dependency problem in semantic segmentation.

Through the analysis of the above semantic segmentation algorithms, it can be seen that the mainstream semantic segmentation algorithms improve the accuracy of segmentation algorithms by using the underlying feature information, extracting multi-scale context information and attention mechanism. [21–23] Therefore, how to find appropriate underlying feature information to help restore images in the process of up-sampling, obtain more semantic information of scale and build appropriate attention mechanism module to enhance the dependency between pixels has become a hot research issue.

Starting from capturing multi-scale semantic information and reusing underlying features, this paper improves the basic residual unit and loss function of the original backbone network model. First, use the multi-level residual structure to obtain more scale semantic information. Secondly, construct a multi-loss function constraint model to use the underlying feature information in the optimization process. Finally, multiple datasets are used to verify the proposed algorithm.

This paper will introduce our proposed method from the five chapters. The first chapter introduces the latest research technology and research status. Chapter II shows the structure and function of ResNet101 and DeepLabV3+. Chapter III presents our method and introduces the experimental process in detail. Then Chapter IV is the experiment of the proposed method, including comparison with other algorithms and some visualization and segmentation results. Finally, Chapter V concludes the contributions and deficiencies of this article.

## 2 Related work

### 2.1 RESNET-101

**Fig 1:**
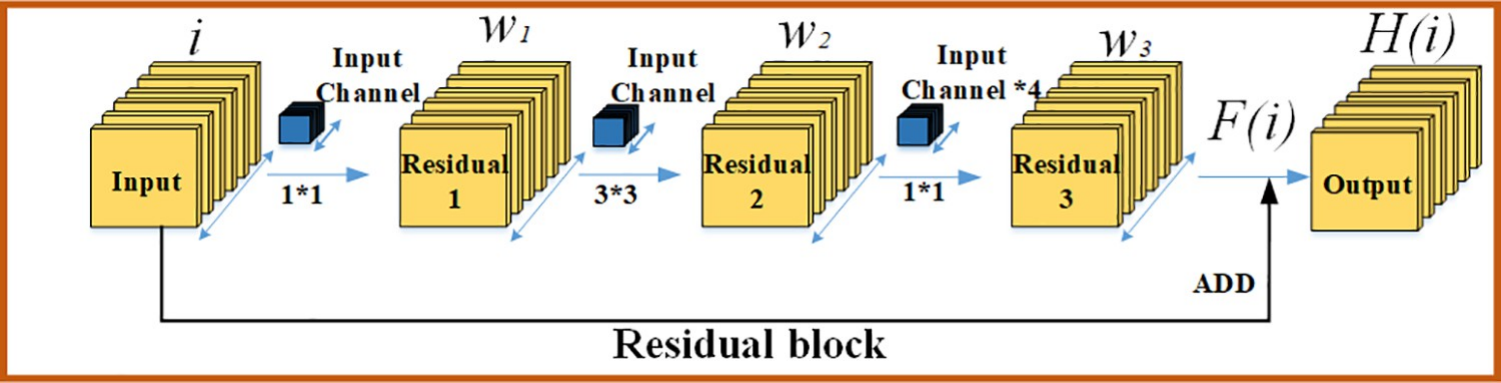
The structure diagram of residual. Residual network uses jump connection to construct residual structure, which solves the degradation problem to some extent.

For traditional convolutional neural networks, it was previously generally believed that the deeper the network was, the stronger the ability of nonlinear expression would be. When a shallow neural network achieves good results in a certain kind of task, more characteristic information can be obtained theoretically by adding a network layer at the back end. However, in fact, with the deepening of the network, the problem of network degradation will occur. Theoretically speaking, if the identity mapping is maintained in the subsequent network layers, that is, when it does not work, the effect of the deep network should be equal to that of the shallow network, and there should be no degradation problem. The reasonable explanation is that the deep network layer is difficult to maintain the identity mapping, resulting in the loss of learning objectives. Therefore, how to make the deep network layer keep the identity mapping is the key factor to solve the degradation problem. The residual structure is shown in Fig 1.

It is assumed that the input of the residual unit is *i*, and the output after passing through three layers of convolutional layer is *F*(*i*). Its definition is shown in Eq (1).


$$F(i)=w_{3}\sigma (w_{2}\sigma (w_{1}i))$$ (1)

In the equation, *w*_1_,*w*_2_,*w*_3_ is the weight parameter of the three-layer convolution layer, and *σ* is the activation function of ReLU. The actual output *H*(*i*) of the residual element is obtained after adding *F*(*i*) to the input *i*, as shown in Eq (2).


$$H(i)=w_{3}\sigma (w_{2}\sigma (w_{1}i))+i$$ (2)

Generally speaking, the one-layer network can be regarded as *y* = *H*(*i*), and the output of each residual unit can be expressed as *H*(*i*) = *F*(*i*)+*i*. In the unit mapping, *y* = *i* is the observed value and *H*(*i*) corresponds to the predicted value, so *F*(*i*) is called the residual. The function of the residual unit is to skillfully convert the learning target into the residual part. As for the neural network, it is obvious that fitting *F*(*i*) = 0 is much easier than fitting *H*(*i*) = *i*, so the depth of the network layer of the residual network can reach far exceeds that of other network structures.

Benefit from the fact that the residual structure can solve the degradation problem caused by the deep network, the depth of the ResNet-101 network can reach 101 layers, and its structure is shown in Table 1. The ResNeT-101 network consists of five parts, which are: Conv1, Conv2_x, Conv3_x, Conv4_x and Conv5_x, where Conv1 is a convolutional layer with step size of 2 and scale of 7×7×64, while Conv2_x-Conv5_x is stacked by residual structure, which contains 3, 4, 23 and 3 residual blocks respectively. Each residual block contains 3 network layers, plus the full connection layer between Conv1 and the end of the network, so there are 101 network layers in total.

**Table 1:** ResNet-101 network structure table.

Layer name	Output size	ResNet-101
Conv1	256×256	7×7, 64, stride 2
		3×3 max pool, stride 2
Conv2_x	128×128	$[\begin{array}{c} 1\times 1,64\\ 3\times 3,64\\ 1\times 1,256 \end{array}]\times 3$
Conv3_x	64×64	$[\begin{array}{c} 1\times 1,128\\ 3\times 3,128\\ 1\times 1,512 \end{array}]\times 4$
Conv4_x	32×32	$[\begin{array}{c} 1\times 1,256\\ 3\times 3,256\\ 1\times 1,1024 \end{array}]\times 23$
Conv5_x	16×16	$[\begin{array}{c} 1\times 1,512\\ 3\times 3,512\\ 1\times 1,2048 \end{array}]\times 3$
Fc	1×1	Average pool
		1000-d, fc, softmax

### 2.2 Deeplabv3+

**Fig 2:**
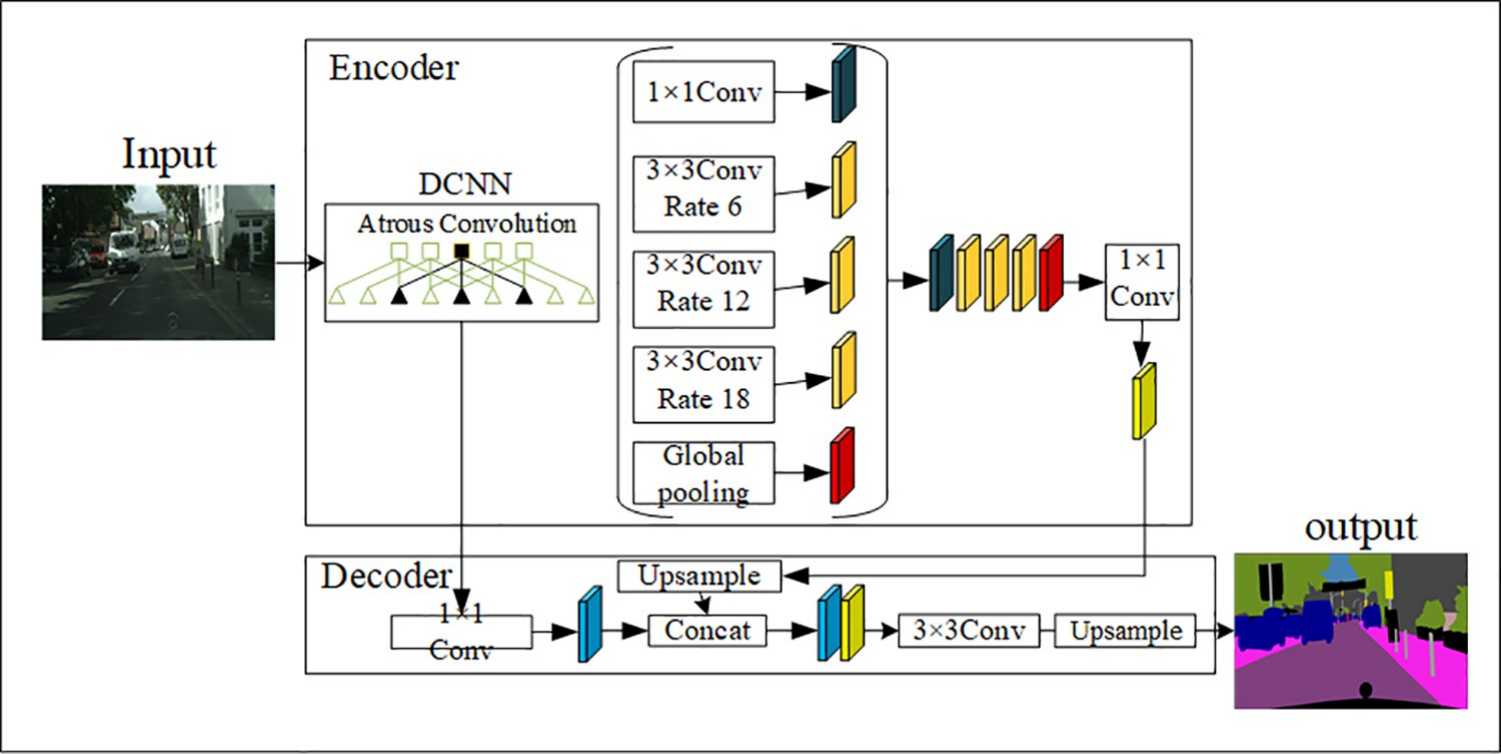
The structure diagram of DeppLabv3+. The Deeplabv3 + adopts residual network as the backbone network, and uses encoding and decoding structure to improve semantic segmentation effect [18].

The structure of Deeplabv3 + [18] is shown in Fig 2.

**Fig 3:**
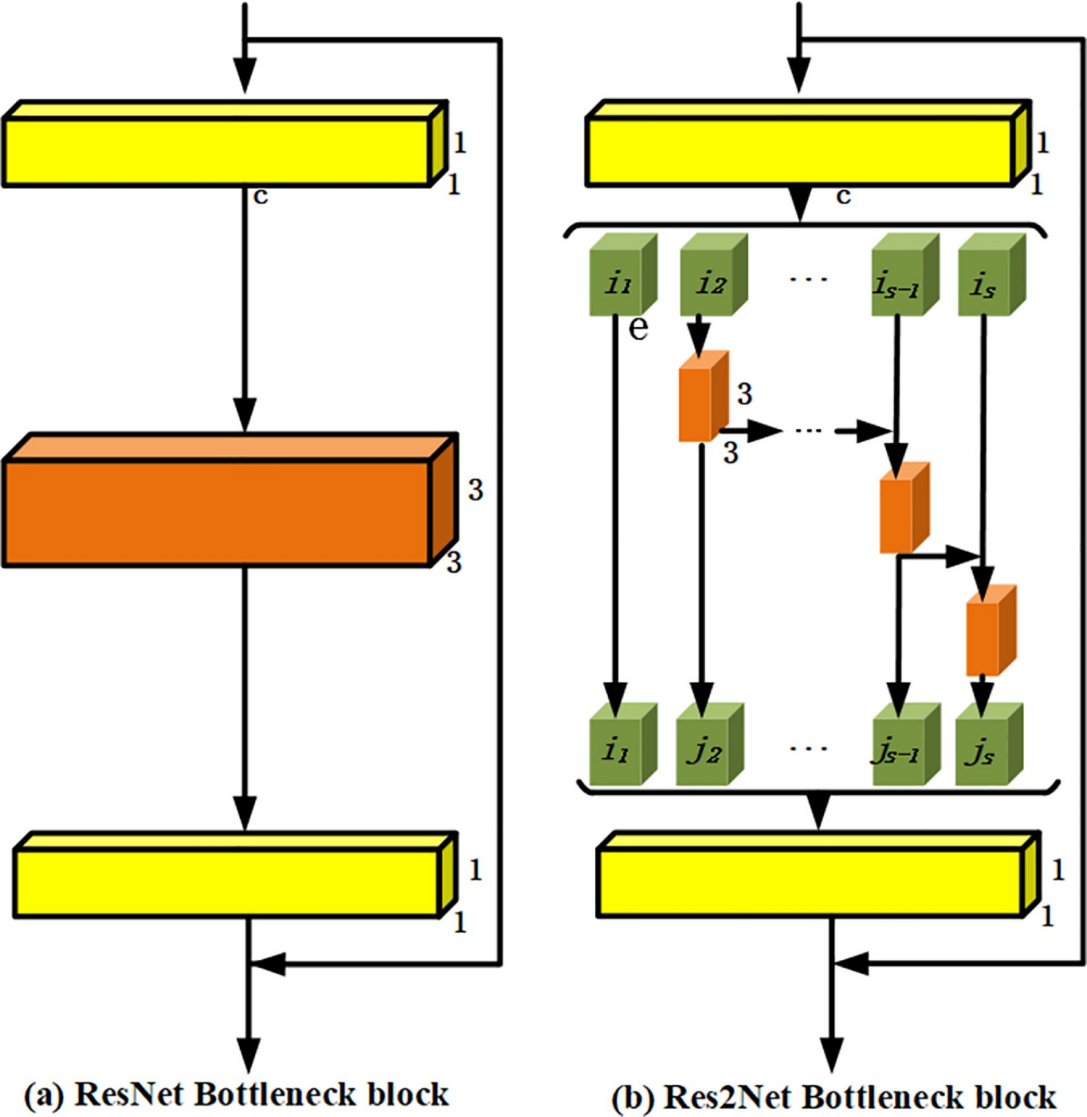
The structure diagram of Res2Net.

Deeplabv3+ encoding structure is composed of ResNet-101 network and void space pyramid module, in which ResNet-101 is mainly divided into 5 parts. The first part is a convolutional layer of 7×7×64, and the other 4 parts contain 3, 4, 23, and 3 blocks respectively. As shown in Fig 3(A), each Block is composed of three convolutional layers plus shortcut connections. The void space pyramid pooling module is connected at the end of the ResNet-101 network to obtain the semantic information of multi-scale images. The decoding structure performs 4-fold bilinear up-sampling on the feature map output by the encoder network, and then merges it with the low-level feature information at the channel level to restore the spatial information lost during the downsampling process.

## 3 The proposed method

### 3.1 Res2net residual cell structure

We use Res2Net structure as the basic unit to construct the backbone network of semantic segmentation framework. As shown in Fig 3, the Res2Net structure on the right constructs a multi-layer similar residue-cascading structure in a single residual unit, which can obtain a larger receptive field range and more scale representation capability compared with the traditional residual unit structure on the left.

The specific operation is as follows:

1. The feature graph output by 1×1 convolution kernel is evenly divided into S feature blocks *i*_1_~*i*_*s*_ in channel dimension;
2. Starting from *i*_2_, the feature block performs concat operation on the number of channels after the next feature block goes through 3×3 convolution. Therefore, the output feature block starting from *j*_2_ contains all the feature information on its left, and with the increase of S, the receptive field range of the output feature graph is increased indirectly.
3. *j*_1_~*j*_*s*_ is spliced and fused, and the number of channels is reduced by 1×1 convolution to get the output feature.

**Fig 4:**
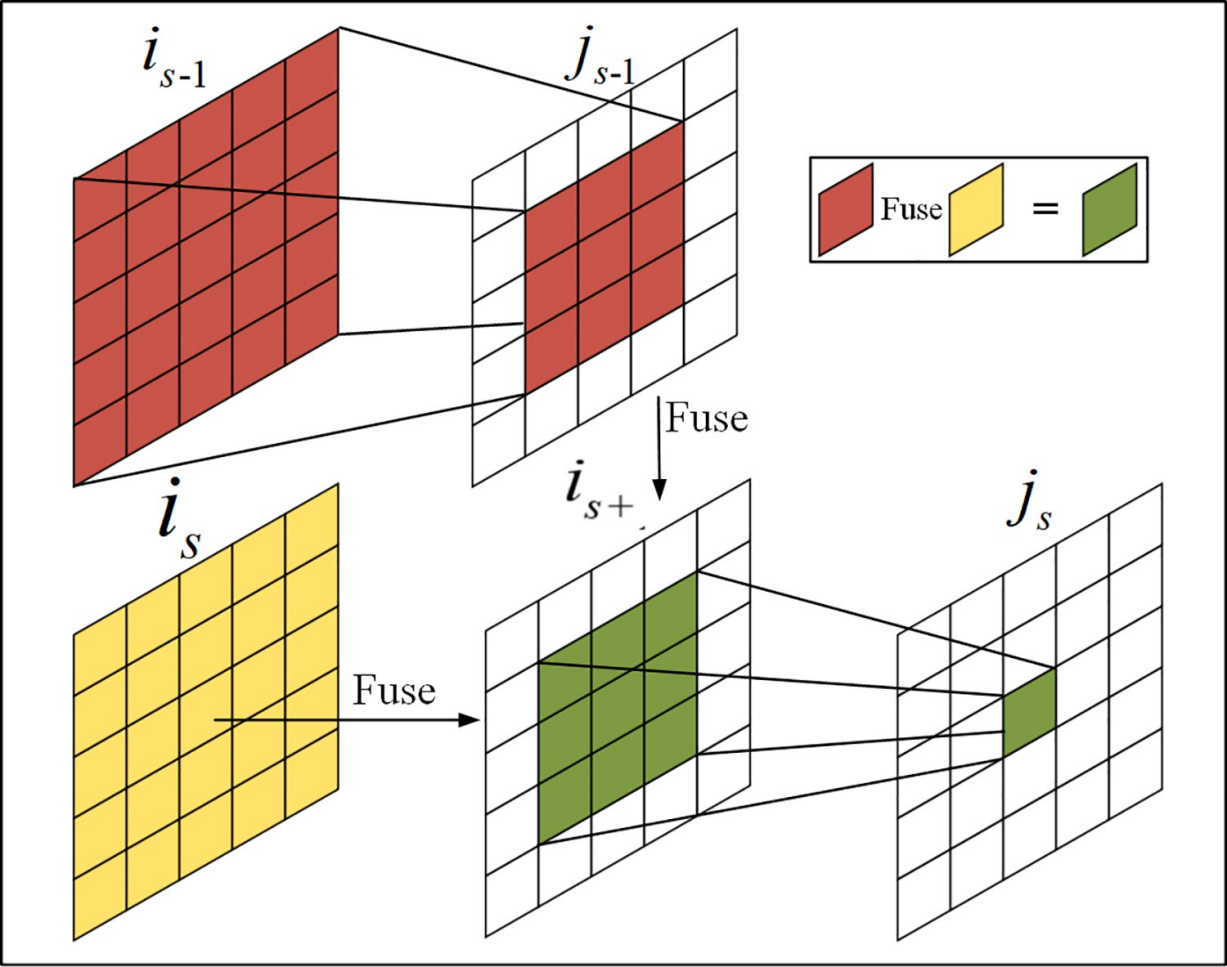
Extended visual field of receptive field.

As can be seen from Fig 4, the reason for the indirect increase of receptive field in feature figure *j*_1_~*j*_*s*_ is as follows:

1. For the feature block *i*_*s*-1_, the feature graph *j*_*s*-1_ is obtained after 3×3 convolution. It is assumed that the receptive field of the pixel on the feature block *j*_*s*-1_ is 3×3.
2. Since the 3×3 convolution is filled, the size of the feature block *j*_*s*-1_ is equal to that of *i*_*s*_, *i*_*s*+_ is obtained after the fusion of the two, and *j*_*s*_ is obtained after the 3×3 convolution of *i*_*s*+_.
3. Analyzing *j*_*s*_ it can be seen from the figure that the receptive field range of the center point of *j*_*s*_ corresponds to the 3×3 Central area of the *i*_*s*+_. The 3×3 region of *i*_*s*+_ center contains the feature information in the 5×5 region of *i*_*s*-1_ feature graph. Therefore, for *j*_*s*_, its receptive field was indirectly expanded to 5×5 area. In addition, the receptive fields of different channel feature maps are different in size, which improves the ability to obtain the features of objects at different scales, so as to obtain more scale semantic features.

### 3.2 Optimization of multi-loss constrained models

**Fig 5:**
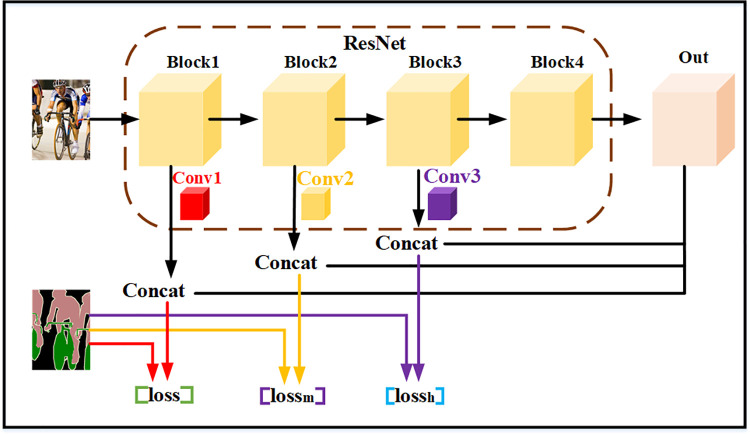
Optimization structure diagram of multi-loss constraint.

In order to make the features of the shallow network gain the semantic segmentation results effectively, this paper establishes several loss functions to constrain the optimization direction of the model. As shown in Fig 5, the feature map Out finally output by the residual network is up-sampled twice and then channel fusion is performed with the feature map Feature map2 output by Block2, and then up-sampling is performed 8 times and the label image is constructed with a middle layer loss. Then use the feature map Feature map3 and the out feature map output by the residual network Block3 to perform channel fusion, and after 16 times up-sampling, build a high-level loss with the label image. Each loss part contains the feature information of different network depths, which helps the model use shallow feature information as much as possible to correct the model parameters in the process of backpropagation.

The weight adaptive method is adopted to assign different weights to different loss parts, and the total loss function *Loss*_*g*_ is obtained as shown in Eq 3.


$$Loss_{g}=Loss_{m}+Loss_{h}+Loss$$ (3)

Where *Loss* is the original loss of the network, *Loss*_*m*_ is the middle-level loss, and *Loss*_*h*_ is the high-level loss. The detailed mathematical expressions of the loss function are shown in Eqs 4 and 5.


$$FL(p_{t})=-{\left(1-p_{t}\right)}^{\gamma }\;\log(p_{t})$$ (4)

$$p_{t}=\{\begin{array}{ll} p & \mathrm{if}\;y=1\\ 1-p & \mathrm{otherwise} \end{array}$$ (5)

### 3.3 Improve the Deeplabv3+ algorithm process

**Fig 6:**
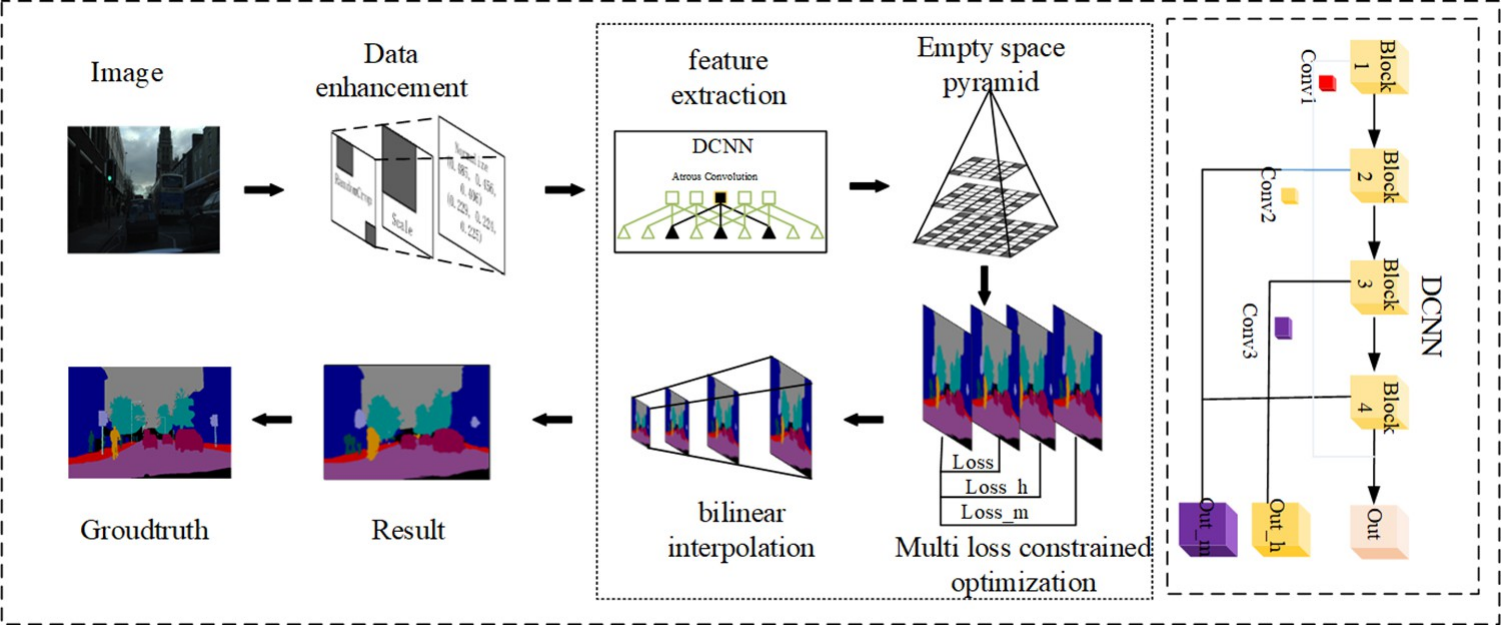
Flow chart of semantic segmentation of urban road images.

Algorithm flow chart shown in Fig 6, we firstly using operations such as standardization, random cutting to deal with data sets. Secondly, the main network was constructed by using multi-level coupling residual structure, and the receptive fields of residual blocks were increased to extract features with multi-scale information. then use of empty space module further extract multiscale pyramid semantic information. Finally, feature images of extracted multi-scale semantic information and network features of different depths were fused to construct multiple loss functions to constrain-model optimization direction, and the prediction results were obtained by up-sampling.

The original algorithm is improved in two directions, namely, obtaining multi-scale semantic information and reusing the underlying features, by using the improved strategies such as multi-level linkage residual structure and multi-loss function constraint, which improves the segmentation accuracy of the model and reduces the occurrence of false segmentation and discontinuous segmentation. The specific algorithm flow is shown as follows.

Step1: Preprocess the training data set by using random cropping, scale transformation, and standard normalization, increase the diversity of training samples, improve the generalization ability of the model, and convert the size of the input image to 513 or 321 according to the type of data.

Step2: Build the backbone network of the model, replace the residual units in the traditional residual network through the multi-level linkage residual structure, so as to obtain more scale feature information, and adjust the number of pooling layers according to the output step size.

Step3: Build the void space pyramid module to process the feature map output by the backbone network, and further obtain the multi-scale semantic information.

Step4: Feature fusion is carried out between the network output feature graphs of different depths in the backbone network and the final network output. After bilinear up-sampling of different degrees, multiple loss functions are constructed with the original label images to constrain the model optimization and realize the reuse of the underlying features.

## 4 Experiment

### 4.1 Datasets and evaluation criteria

In order to verify the effectiveness of the proposed algorithm, experimental data were used from the Cityscapes [24] dataset and CamVid [25, 26] dataset, Cityscapes dataset is divided into 19 categories in semantic segmentation task, including 3475 urban road images with a resolution of 2048*1024, including 2975 training sets and 500 test sets. The CamVid dataset contains 12 semantic categories (including unlabeled categories). There are 701 images in this dataset, including 367 training images, 101 validation images and 233 test images, with a resolution of 480*320 for each image.

In order to evaluate the effectiveness of the proposed algorithm, the average pixel accuracy and average crosswise ratio index were used to evaluate the experimental results. Mean pixel accuracy (mPA) is calculated as shown in Eq 6, which means to calculate the mean value of the proportion of correctly classified pixel points in each class.


$$mPA=\frac{1}{K+1}\frac{\sum_{i=0}^{k}p_{ii}}{\sum_{i=0}^{k}\sum_{j=0}^{k}p_{ij}}$$ (6)

The Intersection over Union (IOU) was calculated to calculate the degree of overlap between the real and predicted values. Its definition is shown in Eq 7. The Mean Intersection over Union (IOU) index was obtained by taking the Intersection over Union index of each class and averaging it. mIoU index intuitively reflects the quality of the segmentation effect.


$$Miou=\frac{1}{K+1}\sum_{i=0}^{k}\frac{p_{ii}}{\sum_{j=0}^{k}p_{ij}+\sum_{j=0}^{k}p_{ji}-p_{ii}}$$ (7)

### 4.2 Experiments settings

The experimental environment is Ubuntu16.04 system, the CPU is Intel-I7-7700, the memory is 16G, the GPU is GeForce GTX 1070, the video memory is 8G, and the deep learning framework is Pytorch 1.4.0. In order to ensure the authenticity of the improved network, the original network and the improved network are set the same hyperparameters, and the control variable method is used to prove the effectiveness of the improved algorithm. Due to the different characteristics of the two sets of data, some parameters of the two sets of data are different as follows: batch_size of Cityscapes dataset is set to 4, and the total number of iterations is 3e4; The CamVid dataset batch size is set to 6, and the total number of iterations is 2e4.

### 4.3 Experimental results and analysis

The Cityscapes dataset contains 19 categories of objects. The overall segmentation accuracy is shown in Table 2, from which it can be seen that the original Deeplabv3 + algorithm can achieve 94.11%, 78.66% and 69.08% for the segmentation of PA, mPA and mIoU index of the Cityscapes dataset.

**Table 2:** Overall segmentation results of Cityscapes (unit: %).

Model	PA	mPA	mIoU
FCN-8s	87.13	41.59	52.81
SegNet	93.82	67.45	59.78
Deeplabv3Plus	94.11	78.66	69.08
Improvement Ⅰ	94.21	80.89	71.08
Improvement Ⅱ	**94.43**	81.91	71.85
Final Algorithm	94.42	**82.40**	**72.15**

It can be seen from the Table 2, when the two improved methods were applied to the segmentation of Cityscapes data, PA、mPA、mIoU index reached 94.42%, 82.40% and 72.15%, increasing by 0.31%, 3.74%, and 3.07% respectively.

After the construction of the multi-loss function constraint model optimization (Improvement Ⅰ), PA, mPA and mIoU are increased by 0.1%, 2.23% and 2% respectively. while after the replacement of the original residual structure by the multi-level linkage residual structure (Improvement Ⅱ), PA, mPA and mIoU indexes are increased by 0.32%, 3.25% and 2.77% respectively.

Table 3 shows the detailed segmentation results of the experiment on the Cityscapes dataset. It can be seen that, for the 19 categories contained in the dataset, the segmentation accuracy of the proposed algorithm in 18 specific categories is higher than that of the original algorithm, especially for the two categories of bus and Fence. The IoU ratio increases by 9.24% and 6%, respectively.

**Table 3:** Segmentation results of Cityscapes dataset experiment (unit: %).

Method Class	Deeplab v3Plus	Improvement Ⅰ	Improvement Ⅱ	Final Algorithm
Road	**96.83**	96.63	96.66	96.71
Sidewalk	**77.94**	77.18	77.53	77.48
Building	88.82	89.44	**89.96**	89.87
Wall	48.57	49.75	**52.79**	51.78
Fence	52.35	55.10	57.06	**58.35**
Pole	46.80	46.37	**50.60**	50.47
Traffic light	53.31	54.50	58.78	**59.12**
Traffic sign	65.31	65.81	67.61	**68.46**
Vegetation	90.08	90.10	**90.52**	90.44
Terrain	58.71	**61.07**	60.23	60.47
Sky	92.48	92.95	93.08	**93.09**
Person	72.53	72.74	74.39	**74.85**
Rider	51.23	53.50	53.82	**55.85**
Car	90.10	90.53	**90.90**	90.72
Truck	68.94	74.71	**77.05**	72.37
Bus	71.66	80.79	78.26	**80.90**
Train	59.62	**70.84**	62.84	67.43
Motorcycle	58.20	58.75	61.98	**62.00**

Table 4 shows the overall segmentation results of this algorithm and its ablation experiment on the CamVid dataset. It can be seen that the pixel accuracy, average pixel accuracy, and mean Intersection over Union index of this algorithm can reach 86.17%, 67.90%, and 55.95%, respectively. Compared with the original algorithm that uses a single improvement strategy, its three segmentation indexes have been improved, especially compared to the original Deeplabv3Plus algorithm, the PA, mPA, and mIoU indexes increased by 0.83%, 5.52%, and 3.59%, respectively.

**Table 4:** Overall segmentation results of CamVid dataset (unit: %).

Model	PA	mPA	mIoU
Deeplabv3Plus	85.34	62.38	52.36
Improvement Ⅰ	85.44	63.21	52.92
Improvement Ⅱ	85.97	65.63	55.01
Deeplabv3Plus+Multiloss+Res2Net	**86.17**	**67.90**	**55.95**

The detailed segmentation accuracy of each category of CamVid is shown in Table 5. It can be seen that among the 12 semantic categories contained in CamVid, the segmentation accuracy of this article is better than the original algorithm in 11 categories, especially in the two categories of Bicyclist and Fence, which are Increased by 10.03% and 10.86% respectively.

**Table 5:** Segmentation results of CamVid dataset experiment (unit: %).

Method Class	Deeplab v3Plus	Improvement Ⅰ	ImprovementⅡ	Final Algorithm
Sky	88.19	**88.25**	87.70	88.05
Building	74.86	**77.68**	75.46	77.17
Pole	15.36	17.66	15.75	**19.80**
Road	89.19	88.13	89.39	**90.07**
Sidewalk	73.06	71.56	73.49	**74.47**
Tree	69.65	**71.75**	68.62	70.60
Sign Symbol	35.00	**38.65**	34.09	37.32
Fence	17.95	**29.07**	21.42	28.81
Car	72.24	**76.81**	71.24	75.92
Pedestrian	38.71	**41.88**	39.17	40.55
Bicyclist	38.54	45.58	42.37	**48.57**
unlabelled	15.59	13.11	16.34	**20.08**

In addition, comparing the segmentation results of the proposed algorithm with the Improved Ⅰ and Improved Ⅱ algorithms in each category, it can be seen that our algorithm has better segmentation results in most categories.

**Fig 7:**
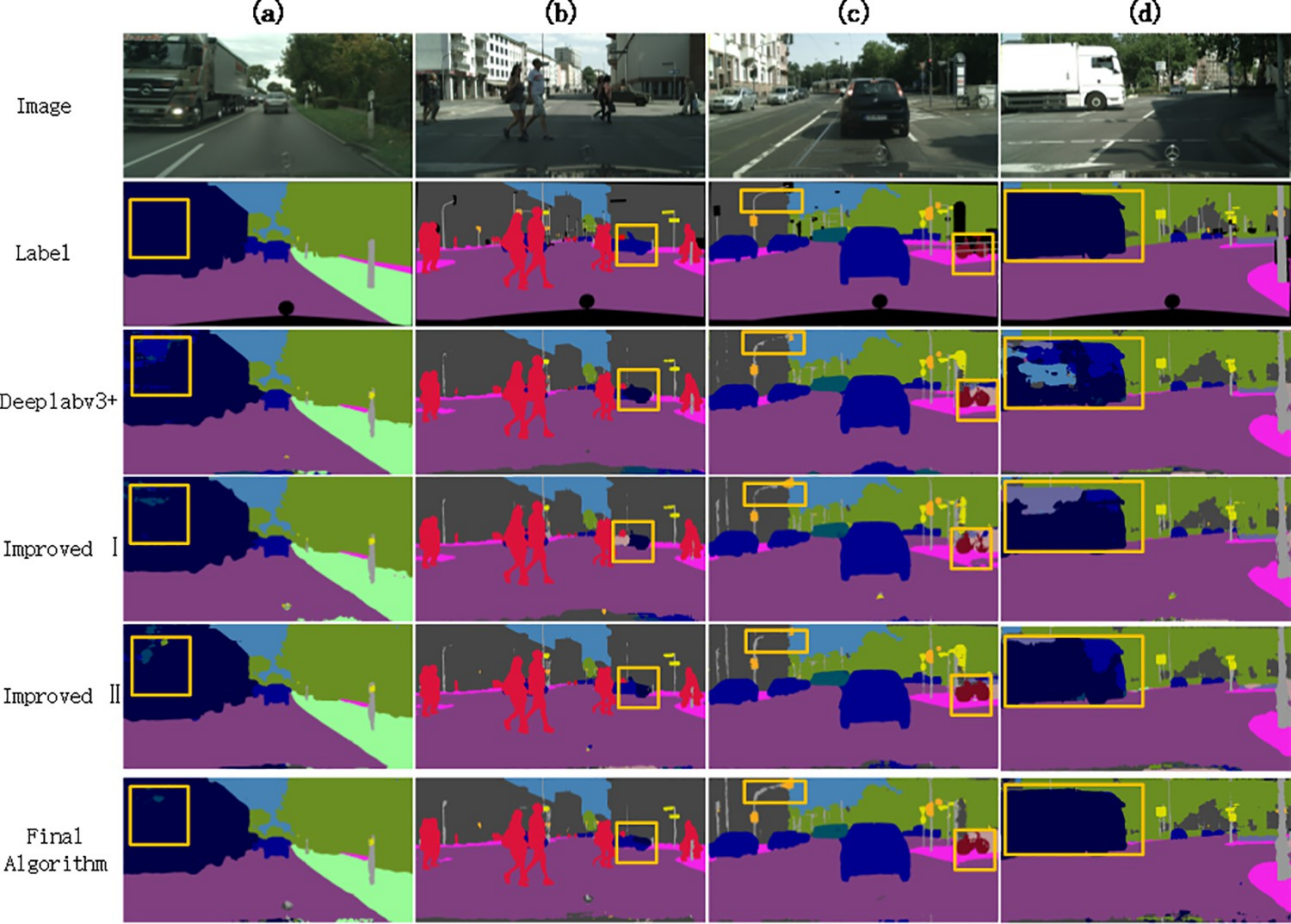
Partial segmentation results of Cityscape dataset. Comparing the first column of images, it can be seen from the labeled area of the yellow box in the figure that the truck head in the original algorithm segmentation result is mis-segmented, and the segmentation results of the Improved Ⅰ and Improved Ⅱ algorithms reduce the mis-segmented area. After applying two innovative methods at the same time, the segmentation area is minimized. In the second column of image labeling area, the original, Improved Ⅰ, and Improved Ⅱ algorithm segmentation results have the phenomenon of mis-segmentation of cars into trucks, and the proposed algorithm is more correct to segment the contours and categories of cars. In the third column of segmentation results, the bicycle contours in the original algorithm and the Improved Ⅰ are relatively fuzzy. In addition, there is a problem of discontinuous segmentation of street lights, and the proposed algorithm can get a clear bicycle contour shape. In the fourth column of images, it can be seen that part of the truck area in the original algorithm was mistakenly divided into cars and sky categories. The Improved Ⅰ and the Improved Ⅱ algorithm reduced the mis- segmented area, and the proposed algorithm segmented the truck area completely and correctly.

Fig 7 is a partial segmentation result diagram of the cityscape, where (a)- (e) are the original label image and the segmentation results corresponding to the 4 sets of ablation experiments.

**Fig 8:**
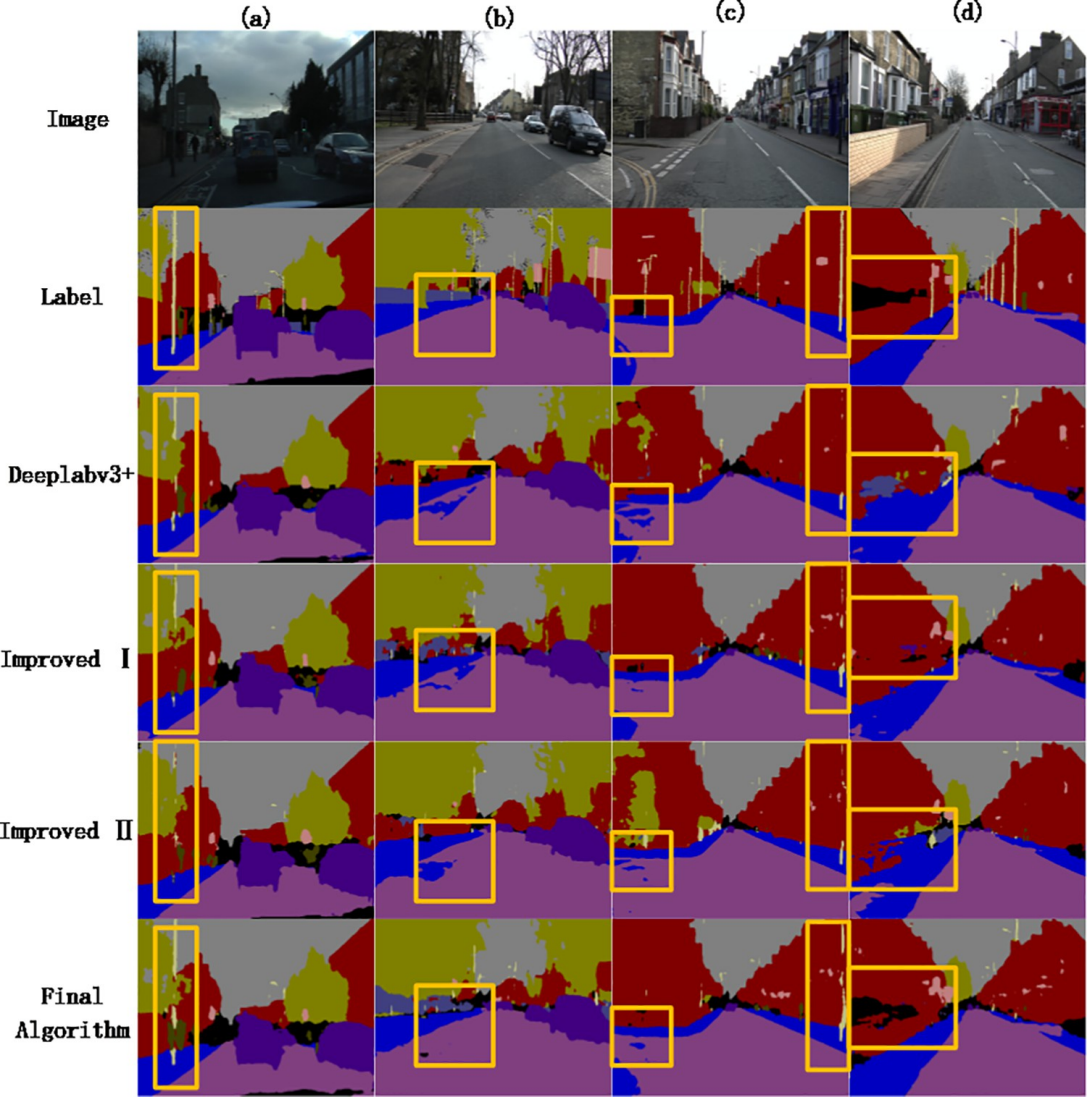
Results of partial segmentation of CamVid dataset. From the image segmentation results in column (a), it can be seen that the Deeplabv3plus algorithm, the Improved Ⅰ and the Improved Ⅱ have different degrees of under-segmentation on the street light poles, and the complete street light pole area cannot be segmented. Compared with the above three methods, the proposed algorithm can segment a more complete street light pole area and get more precise details. In view of the images in column (b) and their segmentation results, Deeplabv3plus, Improved Ⅰ, and Improved Ⅱ algorithms have different degrees of mis-segmentation in the segmentation of the motor vehicle lane area, and part of the motor vehicle lane area is mistakenly divided into sidewalks. In practical applications, the misclassification of these two semantic categories is a fatal error. The algorithm of this paper, which combines two improved strategies, correctly divides the motor vehicle lanes and sidewalks, and solves the mis-segmentation phenomenon. For the images in column (c) and their segmentation results, the sidewalk on the left and the street lights on the right have different degrees of mis-segmentation, especially in the segmentation results of Deeplabv3plus and the improved algorithm. Although the Improved Ⅱ segmented the sidewalk area relatively correctly, the streetlight area marked on the right side was seriously under-segmented. The segmentation result of the algorithm in this paper is still relatively rough compared to the label, but compared with the above segmentation result, the error segmentation phenomenon is reduced. In the image (d) and a series of segmentation results, Deeplabv3plus and the Improved Ⅰ algorithm are not accurate in the segmentation of the marked sidewalk area, while the Improved Ⅱ algorithm and our algorithm obtain more accurate segmentation results.

Fig 8 shows the segmentation results of the algorithm and its ablation comparison test on some CamVid datasets.

## 5 Conclusion

In this paper, an improved Deeplabv3 + algorithm combined with multi-loss constraint model optimization is proposed to solve the problem that the traditional Deeplabv3 + image semantic segmentation algorithm can not reuse the multi-scale feature information and the underlying feature sufficiently. The specific contributions of this paper are summarized as follows:

1. This paper proposes a solution to replace ResNet101 with Res2Net. Compared with the traditional residual unit structure, it can obtain a larger receptive field range and more scale representation ability to solve the problem of poor basic feature extraction ability of the backbone network;
2. This paper innovatively constructs a new loss function composed of multiple loss functions for the network to make the network converge more effectively;
3. This paper combines Res2Net, the new loss function formed by multiple loss functions and the pyramid space module together, and proposes a new more efficient network.

The improvement of the algorithm depends on the multi-layer class residual structure to acquire better features of representation, and the multi-loss function is used to limit the direction of model optimization in the process of back propagation, which causes two problems: first, model training consumes more computing resources and reduces real-time performance. Second, the construction of multiple loss function depends on the quality of artificial design, so it is difficult to ensure the effectiveness of multiple loss function.
